# New Books

**Published:** 2006-11

Air Pollution and Health

Jon Ayres, Robert Maynard, Roy Richards

Hackensack, NJ:World Scientific Publishing, 2006. 264 pp. ISBN: 1-86094-191-5, $66

An Introduction to Molecular Biotechnology: Molecular Fundamentals, Methods and Applications in Modern Biotechnology

Michael Wink, ed.

Hoboken, NJ:John Wiley & Sons, 2006. 828 pp. ISBN: 3-527-31412-1, $100

Chemicals in the Environment

R.M. Harrison, R.E. Hester, eds.

New York:Springer, 2006. 164 pp. ISBN: 0-85404-206-7, $89.95

Finite Mixture and Markov Switching Models

Sylvia Frühwirth-Schnatter

New York:Springer, 2006. 492 pp. ISBN: 0-387-32909-9, $84.95

Health, Hazards and Public Debate: Lessons for Risk Communication from the BSE/CJD Saga

C. Dora

Geneva:World Health Organization Press, 2006. 288 pp. ISBN: 92-8901070-3, $45

Nanocarrier Technologies

M. Reza Mozafari, ed.

New York:Springer, 2006. 225 pp. ISBN: 1-4020-5040-2, $139

Nanoparticulates as Drug Carriers

Vladimir P Torchili, ed.

Hackensack, NJ:World Scientific Publishing, 2006. 880 pp. ISBN: 1-86094-630-5, $148

Organic Pollutants in the Water Cycle: Properties, Occurrence, Analysis and Environmental Relevance of Polar Compounds

Thorsten Reemtsma, Martin Jekel, eds.

Hoboken, NJ:John Wiley & Sons, Inc., 2006. 368 pp. ISBN: 3-527-31297-8, $175

The End of the Wild

Stephen M. Meyer

Cambridge, MA:MIT Press, 2006. 96 pp. ISBN: 0-262-13473-X, $14.95

The Great Lead Water Pipe Disaster

Werner Troesken

Cambridge, MA:MIT Press, 2006. 296 pp. ISBN: 0-262-20167-4, $29.95

The Moral, Social, and Commercial Imperatives of Genetic Testing and Screening

Michela Betta, ed.

New York:Springer, 2006. 268 pp. ISBN: 1-4020-4618-9, $129

The New Environmental Regulation

Daniel J. Fiorino

Cambridge, MA:MIT Press, 2006. 296 pp. ISBN: 0-262-06256-9, $57

